# Rapidly progressive osteoarthritis (RPOA) in companion animals treated with bedinvetmab (Librela™): an expected pathophysiological phenomenon or a cause for concern?

**DOI:** 10.3389/fvets.2025.1640217

**Published:** 2025-08-29

**Authors:** Ali Mobasheri, Peter Hanson, Jonathan Larkin

**Affiliations:** ^1^Research Unit of Health Sciences and Technology, Faculty of Medicine, University of Oulu, Oulu, Finland; ^2^Department of Regenerative Medicine, State Research Institute Centre for Innovative Medicine, Vilnius, Lithuania; ^3^Department of Joint Surgery, First Affiliated Hospital of Sun Yat-Sen University, Guangzhou, China; ^4^Image Analysis Group (IAG), London, United Kingdom; ^5^Spruce Biotech Consulting, Beverly, MA, United States; ^6^SynOA Therapeutics, Philadelphia, PA, United States

**Keywords:** bedinvetmab, osteoarthritis, rapidly progressive osteoarthritis (RPOA), nerve growth factor, anti-NGF therapy, pharmacovigilance, pain, drug safety

The advent of anti-nerve growth factor (NGF) monoclonal antibodies, such as bedinvetmab (Librela™), has marked a significant advancement in the management of osteoarthritis (OA) pain in dogs (1, 2). First approved for use in October 2020 (EU) and May 2023 (U.S.), bedinvetmab offers a novel mechanism of action by targeting NGF, a key mediator in pain pathways, thereby providing relief for canine OA patients (1, 3).

While clinical trials have demonstrated the efficacy and safety of bedinvetmab, post-marketing surveillance has brought to light reports of adverse events, including neurological signs such as ataxia, seizures, and paresis, as well as musculoskeletal issues like lameness (4). Similar observations have been made in humans with OA treated with NGF neutralizing antibodies such as Tanezumab (5), which was originally developed for the treatment of human OA (6). These observations have collectively raised concerns about the potential for rapidly progressive OA (RPOA) in dogs undergoing treatment with bedinvetmab (4) and humans receiving Tanezumab (7).

The term RPOA has recently become a focal point in OA drug development, particularly following its association with the anti–NGF class of analgesics (8). RPOA is characterized by an accelerated deterioration of joint structures, leading to severe pain and functional impairment. In human medicine, RPOA has been associated with anti-NGF therapies (9, 10), particularly when used concomitantly with nonsteroidal anti-inflammatory drugs (NSAIDs). The pathophysiology is thought to involve the suppression of pain signals, resulting in increased joint usage and subsequent structural damage (5). In veterinary medicine, the occurrence of RPOA in dogs treated with bedinvetmab remains a subject of investigation, monitoring and scrutiny. Recent case reports have reported rapid joint deterioration in dogs with elbow OA, following the initiation of bedinvetmab therapy. While case reports do not establish causality, they underscore the need for vigilance and further safety monitoring. The potential for RPOA in canine patients necessitates a cautious approach, especially regarding the concurrent use of NSAIDs, despite previous reports of acceptable safety (3). Although the safety of combining bedinvetmab with NSAIDs has not been fully established, some practitioners have employed short-term NSAID therapy during the initiation of bedinvetmab treatment. Given the experiences in human medicine, it may be prudent to limit or closely monitor such combinations until more definitive data are available.

The identification of RPOA in dogs is unfortunately complicated by the lack of standardized diagnostic criteria and the variability in clinical presentations. Advanced imaging modalities and longitudinal structural studies are essential to differentiate RPOA from the natural progression of OA and to elucidate any potential links to anti-NGF therapies. This reminds us of the crucially important work that was carried out by the Nobel Prize winner Dr. Rita Levi-Montalcini^1^ and her colleagues almost 30 years ago, following the discovery of NGF (11), warning that NGF must be viewed as a multifactorial mediator that modulates more than pain responses, and is involved in neuroimmune-endocrine functions of vital importance to the regulation of physiological homeostatis, with potential involvement in pathological processes deriving from dysregulation of either local or systemic homeostatic balances (12). Any treatment that blocks the production and the signal of this vital growth factor requires long-term safety monitoring in humans and in companion animals.

In conclusion, while bedinvetmab represents a promising option for managing OA pain in dogs, the emergence of adverse events resembling RPOA warrants careful consideration. Veterinarians should remain alert to signs of rapid joint deterioration in companion animals receiving bedinvetmab and they must report such cases to appropriate pharmacovigilance systems. Case reports are generally regarded as week in terms of scientific and medical evidence and we need better processes for integrating them into scientific and medical knowledge. The number of adverse cases associated with bedinvetmab use are generally low, but they do occur. Therefore, further research is imperative to determine the incidence, risk factors, and pathogenesis of RPOA in canine patients, thereby ensuring the safe and effective use of anti-NGF therapies in veterinary practice.

## Footnotes

^1^ https://www.nobelprize.org/stories/women-who-changed-science/rita-levi-montalcini/
